# Correlation Analysis of Prognostic Gene Expression, Tumor Microenvironment, and Tumor-Infiltrating Immune Cells in Ovarian Cancer

**DOI:** 10.1155/2023/9672158

**Published:** 2023-10-06

**Authors:** Qing Li, Zongjing Yang, Xingqing Ling, Junming Ye, Jiaying Wu, Yu Wang, Chun Yao, Jinghui Zheng

**Affiliations:** ^1^Guangxi University of Chinese Medicine, Nanning 530000, Guangxi, China; ^2^Department of Geriatrics, Ruikang Hospital Affiliated to Guangxi University of Chinese Medicine, Nanning 530011, Guangxi, China; ^3^Ruikang Hospital Affiliated to Guangxi University of Chinese Medicine, Nanning 530011, Guangxi, China

## Abstract

**Objective:**

Tumor microenvironment (TME) research can provide a crucial direction for the innovation and continuous improvement of novel biologic therapies for cancer. This study examined the relationship between the TME, expression profiles of the tumor-infiltrating immune cell, and prognostic gene expression in ovarian cancer (OC).

**Materials and Methods:**

Screening of CD3E, CD3G, CD2, CD3D, CCL19, and IL2RG was performed using the bioinformatics methods.

**Results:**

All six genes were found to participate in immune-related molecular mechanisms and could regulate the expression of tumor-infiltrating cells. A Kaplan–Meier survival analysis results demonstrated a strong association between overall survival and all gene expressions in patients with OC. CIBERSORT analysis results showed that the expression level of all genes was positively correlated with *γδ* T cell proportions.

**Conclusion:**

Therefore, in the OC microenvironment, CD3E, CD3G, CD2, CD3D, CCL19, and IL2RG can be potential immunotherapy targets and prognostic markers.

## 1. Introduction

Ovarian cancer (OC) is a malignant tumor of the female reproductive system, and its incidence and mortality rank top 10 in the global cancer statistics report [1]. OC is almost asymptomatic in the early stage. Further, in most cases, treatment, particularly surgery supplemented with chemical drug therapy, radiotherapy, immunotherapy, and targeted therapy, is provided at the late stage. Among the different types of treatment options, chemical drug therapy has evident outcomes. However, it is significantly associated with drug resistance and side effects, and its curative effect on patients with recurrent and advanced-stage OC is limited [2, 3]. Nevertheless, the surrounding microenvironment has an impact on tumor progression. The tumor microenvironment (TME) contains tumor cells and tumor-related matrices, such as stromal cells, tumor-infiltrating immune cells (TICs), and fibroblasts [4]. The studies by Stanton and Disis [5] and Sadozai et al. [6] suggest that TICs may affect breast cancer, melanoma, and other tumor patients' clinical outcomes in terms of patient prognosis. However, the state of TICs in the OC microenvironment and their role in the prognosis of patients with OC should be further explored.

The efficacy of immunotherapy, which is a novel treatment, is limited. However, it is still one of the most promising therapies [7]. Adoptive cell therapy (ACT) and immune checkpoint blockade (ICB) therapy are particularly effective against malignant melanoma, gastric cancer, and non-small cell lung cancer [8, 9]. Immunotherapy is still being studied for its potential therapeutic benefits and uses in OC.

In a previous study, the ratio of stromal and immune components in OC samples from The Cancer Genome Atlas (TCGA), as well as the proportion of TICs, were calculated using the Estimation of STromal and Immune cells in MAlignant Tumor tissues using Expression (ESTIMATE) data [10] and CIBERSORT [11] algorithms. Prognostic biomarkers that can predict immunotherapy outcomes and OC prognosis were also identified.

## 2. Materials and Methods

### 2.1. Datasets Collected from TCGA

We downloaded transcriptome RNA sequencing data from TCGA (https://portal.gdc.cancer.gov/) from 379 patients with OC tumor samples. Next, clinical information, including overall survival time and survival status was extracted.

### 2.2. Use of the ESTIMATE Algorithm and Detection of the Immune and Stromal Groups

The ESTIMATE algorithm identified stromal and immune microenvironment infiltration using gene expression data. The analytic approach was integrated into R 4.1.2's estimate package, and the expression profiles of two distinct sets of 141 genes showed the degree of tumor immune and stromal infiltrations. Thus, stromal and immune scores in TCGA-OV samples were calculated based on expression matrices retrieved from the RNA sequencing data. The ESTIMATE score, which is the sum of a patient's stromal and immune scores, represents tumor purity. The tumor purity was lower when the ESTIMATE score was higher.

### 2.3. Correlation between ICB Treatment Response and TME

The tumor immune dysfunction and exclusion (TIDE) algorithm uses gene expression patterns to predict tumor ICB therapy response. Using gene expression markers, the TIDE algorithm estimates two tumor immune evasion mechanisms: immunosuppressive factors mediated cytotoxic T lymphocyte (CTL) exclusion and the dysfunction of tumor infiltration CTLs. Antitumor immune escape is more likely in patients with higher TIDE scores. Hence, they respond less to ICB therapy. The potential response of the OC samples to the ICB therapy was assessed using the TIDE algorithm. The unpaired *t*-test was utilized to compare TIDE scores, especially stromal and immune scores, between high and low subgroups to determine if TME and ICB treatment were related. Median immune and stromal scores were cutoffs for high- and low-score groups. TIDE and immune/stromal scores were correlated using Spearman correlation coefficients.

### 2.4. Differentially Expressed Genes Identification

Differentially expressed genes (DEGs) were screened between high- and low-score groups by means of the Wilcoxon rank-sum test, and the limma R package was used to organize and cluster the DEGs. As screening criteria, a |log2 (Fold Change)| >1 and false discovery rate (FDR) < 0.05 were used. The VennDiagram package was used to obtain the intersected DEGs between the stromal and immune scores.

### 2.5. Functional Enrichment, Protein–Protein Interaction (PPI) Network, and Pathway Analysis of DEGs

The clusterProfiler [12] package was used to perform gene ontology (GO) enrichment and Kyoto Encyclopedia of Genes and Genomes (KEGG) pathway analyses (*p* < 0.05). A statistically significant FDR was <0.05. The PPI network was developed utilizing STRING (https://cn.string-db.org/) data to better comprehend the relationship among the screened genes. A minimum necessary interaction score of high confidence (0.95) was selected. Cytoscape (version 3.9.1) was then used to visualize the PPI network. Subsequently, CytoNCA, a Cytoscape plugin for analyzing the centrality of PPI networks, was used to identify the network's crucial genes. The crucial genes were chosen based on their degree of centrality. Crucial genes were defined as genes with centrality values greater than two times the median centrality value in the PPI network.

### 2.6. Predictive Value of Crucial Genes in Survival Analysis

Kaplan–Meier (K–M) and univariate Cox proportional hazards regression analyses were carried out with the aid of the survival package to examine the prognostic value of differentially expressed TME-related genes in patients with OC. Only genes that had a *p*-value < 0.05 were regarded as prognostic genes. The intersected crucial prognostic genes were identified among the crucial genes and prognostic genes based on the VennDiagram package. The median crucial prognostic gene expression divided patients into distinct groups. Using R's survival package, a K–M analysis compared overall survival between low- and high-expression groups.

### 2.7. Gene Set Enrichment Analysis (GSEA)

We divided the TCGA-OV dataset samples into two groups based on median crucial prognostic gene expression to better understand the mechanisms. Next, GSEA (https://www.gsea-msigdb.org/gsea/index.jsp) was conducted to examine if the two groups' genes were rich in relevant biological processes. The annotated gene set c2.cp.kegg. v7.5.1. symbols. The reference gene set Gmt was selected. The statistical significance criteria were set as an FDR < 0.05 and *p*-value < 0.05.

### 2.8. Correlation between TICs and Crucial Prognostic Gene Expression

The TCGA-OV dataset's normalized gene expression data using the CIBERSORT algorithm (https://cibersort.stanford.edu/) estimated 22 TIC subtype proportions. Only patients with CIBERSORT *p*-values < 0.05 were included in the subsequent analyses. The estimated immune cell type fractions for each sample were 1. Based on crucial prognostic gene expression in OC patients, the Wilcoxon rank-sum test was utilized to compare TIC proportions in low- and high-expression groups. A *p*-value < 0.05 was significant. TIC content and crucial prognostic gene expression were assessed to be correlated using Pearson correlation coefficients.

## 3. Results

### 3.1. Correlation between TME and ICB Treatment Response

**Figure 1:**
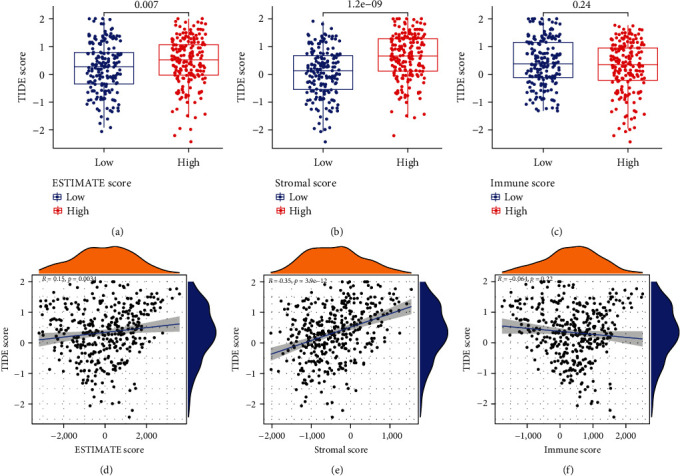
(a–c) Differences between the low- and high-score groups in terms of the ESTIMATE, immune, and stromal scores. (d–f) Association between the TIDE and ESTIMATE scores, and the stromal and immune score based on the Spearman correlation analysis.

ESTIMATE algorithm-generated stromal, ESTIMATE, and immune scores were strongly positively linked to the TIDE score (*p* < 0.01). According to the TIDE score, patients with a low-stromal cell infiltration and a high-tumor purity may be more sensitive to ICB therapy (Figure 1).

### 3.2. DEGs between Stromal and Immune Scores

**Figure 2:**
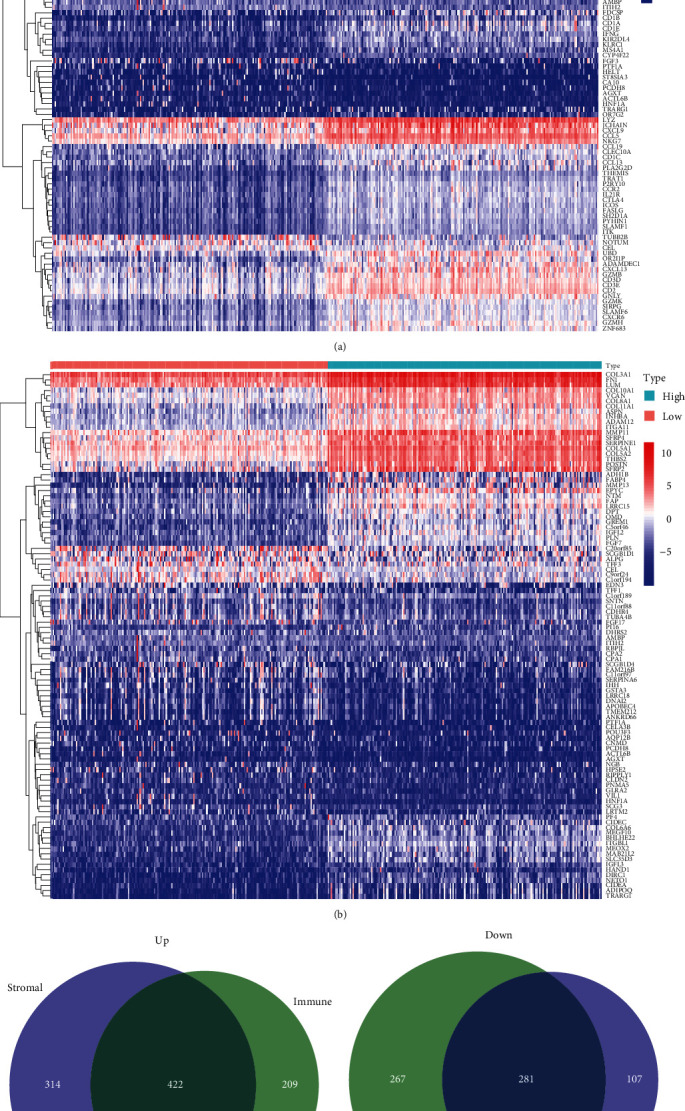
(a and b) Each heatmap showed the top 50 upregulated and downregulated DEGs based on absolute values logFC. (c and d) Venn plots illustrating the 422 upregulated and 281 downregulated DEGs most frequently associated with immune and stromal scores.

Heatmaps revealed distinct gene expression profiles between groups with high and low stromal and immune scores. Based on immune scores, we identified 1,124 DEGs, including 736 upregulated and 388 downregulated genes. Similarly, based on the immune scores, 1,179 DEGs were identified, including 631 upregulated and 548 downregulated genes. In both the stromal and immune score groups, effective DEGs overlapped genes, and 703 common DEGs were discovered, including 422 upregulated and 281 downregulated genes (Figure 2).

### 3.3. DEG Enrichment Analysis of GO Function and KEGG Pathways

**Figure 3:**
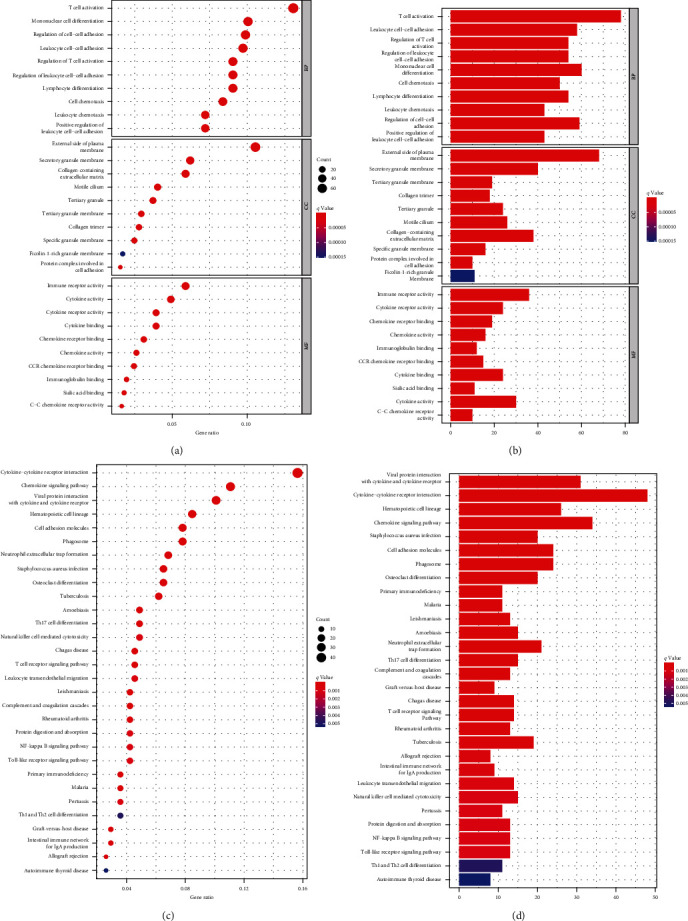
(a–d) Top 30 terms in GO and KEGG based on gene enrichment score and *q*-value.

Additionally, 703 DEGs were enriched in three GO categories. Activating T cells, plasma membrane outside, and immune receptor activity were the most critical factors in biological process (BP), cell component (CC), and molecular function (MF) categories, respectively. Cytokine–cytokine receptor interaction, viral protein–cytokine interaction, and chemokine signaling pathway were the top three KEGG terms in 703 DEGs as per enrichment analysis results. KEGG and GO enrichment analyses predicted DEGs' involvement with immune-related activities (Figure 3).

### 3.4. The PPI Network and Cox Proportional Hazards Regression Analysis Identified Six Crucial Prognostic Genes in OCA

**Figure 4:**
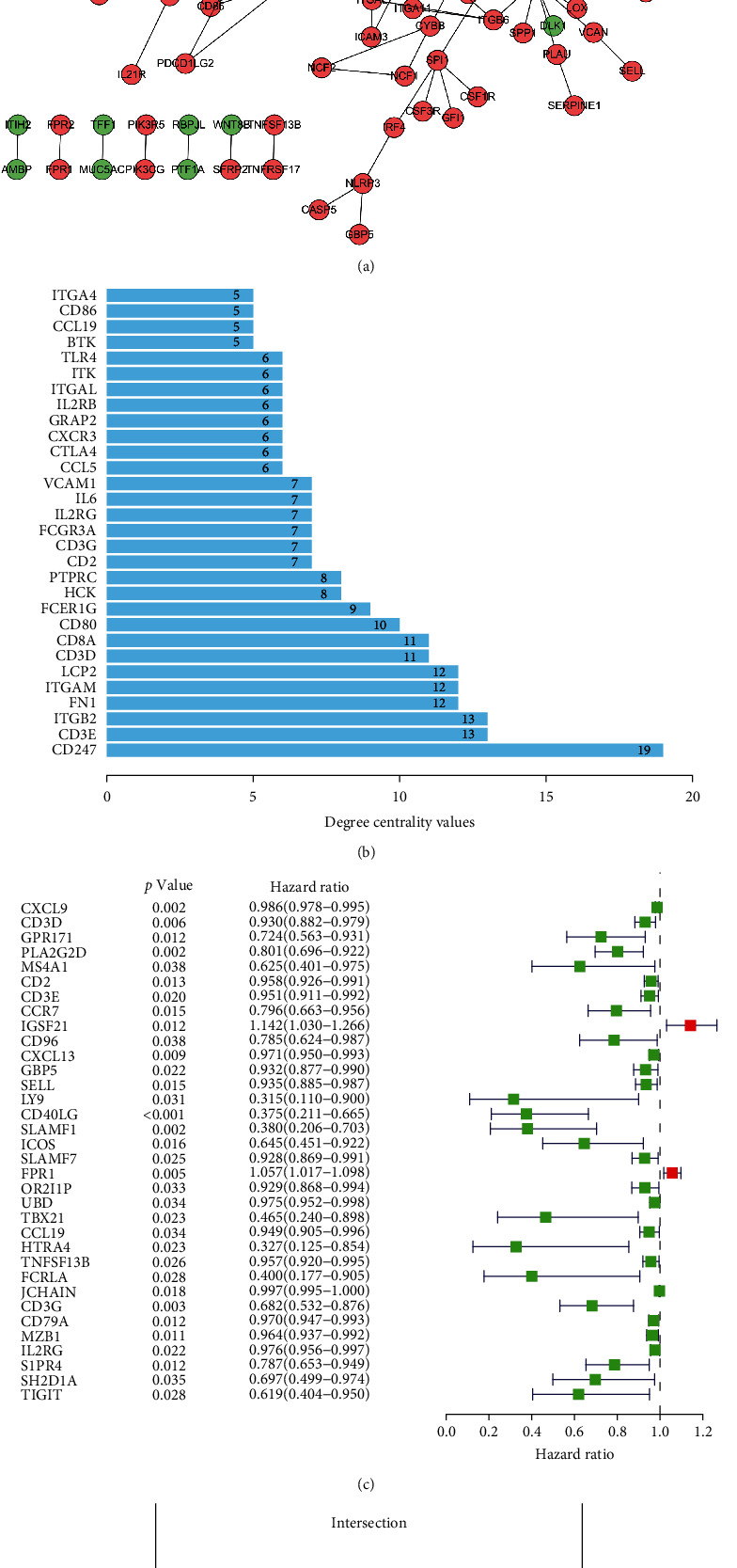
(a) The interacting PPI network comprised 148 genes. (b) The bar plot represented the top 30 crucial genes screened according to the centrality values. (c) DEG-related univariate Cox proportional hazards regression analysis results are displayed on the forest map. (d) Venn plot showing six crucial prognostic genes.

Cytoscape was used to develop a STRING database-based PPI network to evaluate if the 703 DEGs have protein interactions. A PPI network with a 0.95 minimum interaction score was developed using the 148 genes. According to the degree centrality values in the bar plot, the top 30 crucial genes were screened. The forest map depicted the 34 prognostic TME-related genes identified through K–M (*p* < 0.05) and univariate Cox proportional hazards regression (*p* < 0.05) analyses of 703 DEGs. Meanwhile, the top 34 genes in the univariate Cox proportional hazards regression analysis and the top 30 crucial genes for the degree centrality values in the PPI network intersected the CD3E, CD2, CD3D, CD3G, IL2RG, and CCL19, respectively (Figure 4).

### 3.5. Correlation between Survival and Six Crucial Prognostic Genes Expression

**Figure 5:**
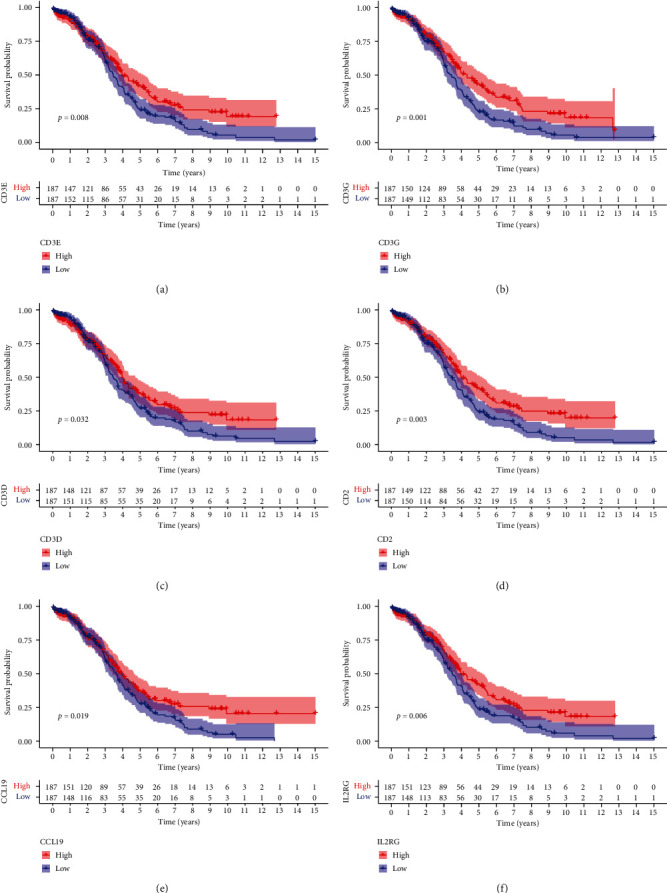
(a) The link between survival rate and CD3E expression was examined using Kaplan–Meier (K–M) analysis. (b) The link between survival rate and CD3G expression was examined using K–M analysis. (c) The link between survival rate and CD3D was examined using K–M analysis. (d) The link between survival rate and CD2 was examined using K–M analysis. (e) The link between survival rate and CCL19 was examined using K–M analysis. (f) The link between survival rate and IL2RG expression was examined using K–M analysis.

To investigate the correlation between gene expression and survival, all OC samples were divided into high- and low-expression groups as per the six crucial prognostic genes' median expression levels. The findings demonstrated a favorable correlation between highly crucial prognostic gene expression and survival (Figure 5).

### 3.6. GSEA

**Figure 6:**
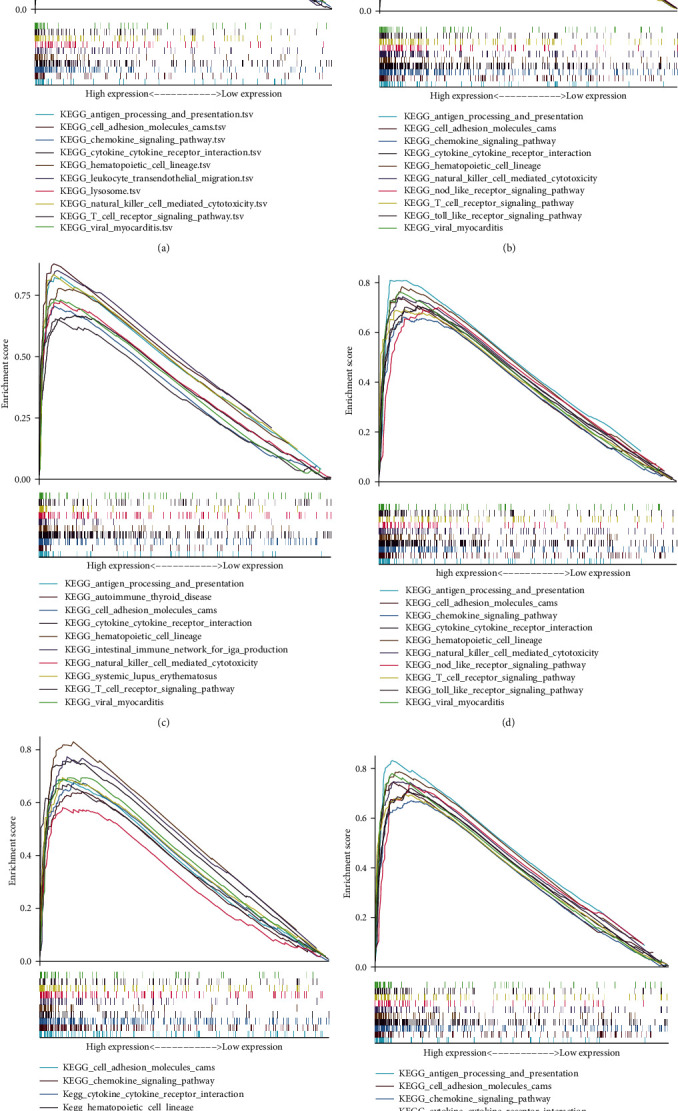
(a) GSEA identified the top 10 immune pathway signatures remarkably enriched in CD3E and have a high-phenotypic expression. (b) GSEA identified the top 10 immune pathway signatures remarkably enriched in CD3D and have a high-phenotypic expression. (c) GSEA identified the top 10 immune pathway signatures remarkably enriched in CD3G and have a high phenotypic expression. (d) GSEA identified the top 10 immune pathway signatures remarkably enriched in CD2 and have a high-phenotypic expression. (e) GSEA identified the top 10 immune pathway signatures remarkably enriched in CCL19 and have a high-phenotypic expression. (f) GSEA identified the top 10 immune pathway signatures remarkably enriched in IL2RG and have a high-phenotypic expression.

GSEA identified differentially expressed signaling pathways between high- and low-crucial prognostic gene expression groups in OC. The MSigDB Collection (c2.cp.kegg. v7.5.1. symbols) enrichment analysis results showed significant variations (FDR < 0.05). The signaling pathways that were most significantly enriched were chosen using a normalized enrichment score. Results showed that cell adhesion molecules, natural killer (NK) cell-mediated cytotoxicity, NOD-like receptor signaling pathway, hematopoietic cell lineage, T cell receptor signaling pathway, cytokine–cytokine receptor interaction, chemokine signaling pathway, Toll-like receptor signaling pathway, and antigen processing and presentation were enriched in the crucial prognostic genes with a high-expression phenotype (Figure 6).

### 3.7. Correlation of TICs and Six Crucial Prognostic Genes Expression

**Figure 7:**
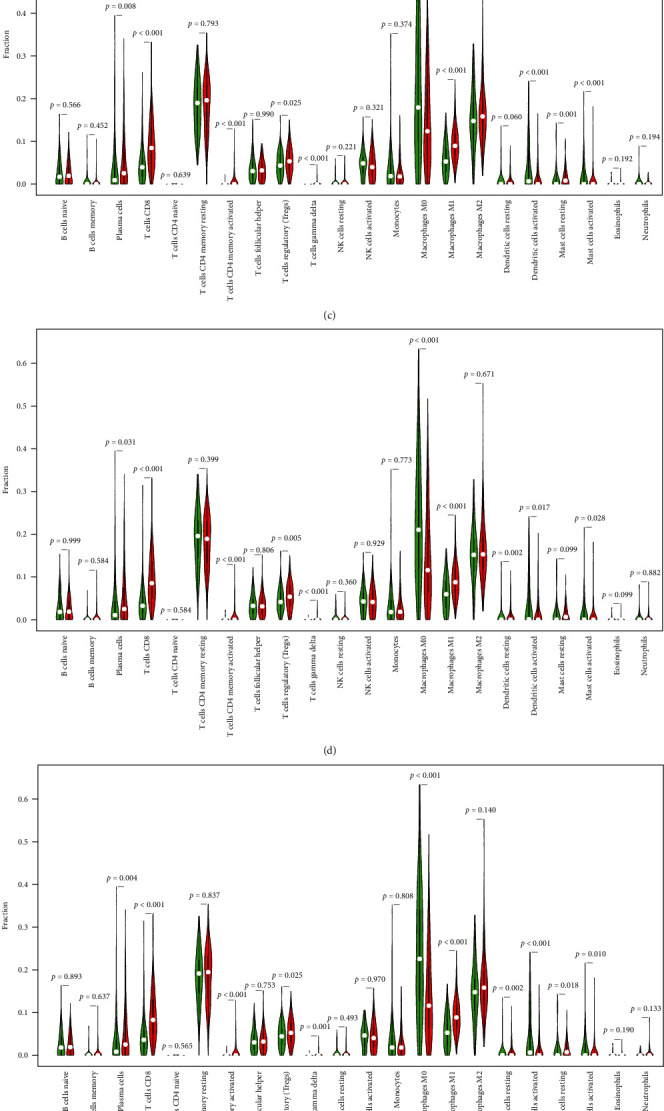
(a) A heatmap depicting the relationship between 22 different types of TICs. Each tiny colored box's shade represented the two cells' correlation value. For significance testing, the Pearson coefficient was used. (b) Using the Wilcoxon rank-sum test, violin plots compare the percentages of 22 distinct immune cell types expressing low (green) or high (red) levels of CD3E in tumor tissues. (c) Using the Wilcoxon rank-sum test, violin plots compare the percentages of 22 distinct immune cell types expressing low (green) or high (red) levels of CD3G in tumor tissues. (d) By means of the Wilcoxon rank-sum test, violin plots compare the percentages of 22 distinct immune cell types expressing low (green) or high (red) levels of CD3D in tumor tissues. (e) By means of the Wilcoxon rank-sum test, violin plots compare the percentages of 22 distinct immune cell types expressing low (green) or high (red) levels of CD2 in tumor tissues. (f) By means of the Wilcoxon rank-sum test, violin plots compare the percentages of 22 distinct immune cell types expressing low (green) or high (red) levels of CCL19 in tumor tissues. (g) By means of the Wilcoxon rank-sum test, violin plots compare the percentages of 22 distinct immune cell types expressing low (green) or high (red) levels of IL2RG in tumor tissues.

The CIBERSORT algorithm in R 4.1.2 and limma assessed each OC sample's TME immune cell ratios to find the correlation between six crucial prognostic genes expression and TICs. Results showed that immune cells linked positively to activated memory CD4+ T cells and CD8+ T cells (*r* = 0.35) and negatively with macrophage M0 and monocytes (*r* = 0.43) (Figure 7).

Then, correlation and difference analyses showed that CD3E expression was strongly correlated with 11 TIC types. Results showed that resting hypertrophy cells, activated memory CD4+ T cells, macrophage M1, regulatory T cells, CD8+ T cells, resting dendritic cells, and *γδ* T cells were positively linked to the CD3E expression. CD3E expression was negatively linked to macrophages M0, eosinophils, and activated mast and dendritic cells.

Correlation and difference analyses revealed that CD3D expression was strongly associated with 10 TIC types. Results showed that plasma cells, activated mast cells, regulatory T cells, activated dendrites cells, resting dendritic cells, and *γδ* T cells were positively associated with CD3D expression. CD3D expression was negatively linked to activated memory CD4+ T cells, macrophage M1, CD8+ T cells, and macrophage M0.

Correlation and difference analyses revealed that CD3G expression was significantly correlated with 10 TIC types. Results showed that CD3G expression was positively linked to plasma cells, activated dendritic cells, resting mast cells, *γδ* T cells, regulatory T cells, and macrophage M0. CD3G expression was negatively linked to CD8+ T cells, macrophage M1, and activated memory CD4+ T cells.

Correlation and difference analyses revealed that CD2 expression was significantly correlated with 11 types of TIC. Results showed that CD2 expression was positively linked to plasma cells, active dendritic cells, regulatory T cells, resting dendrites, and T cells. CD2 expression was negatively linked to macrophage M0, CD8+ T cells, macrophage M1, and activated memory CD4+ T cells.

Correlation and difference analyses revealed that CCL19 expression was significantly correlated with 10 types of TIC. Results showed that activated mast cells, activated dendritic cells, naive B cells, *γδ* T cells, activated NK cells, and activated memory CD4+ T cells were positively associated with CCL19 expression. CD8+ T cells, macrophage M1, resting dendritic cells, and plasma cells had negative correlations with CCL19 expression.

Correlation and difference analyses showed that 10 TIC types substantially correlated with IL2RG expression. Plasma cells, *γδ* T cells, regulatory T cells, eosinophils, macrophage M0, resting dendritic cells, and active dendritic cells were found to be positively correlated with IL2RG expression. Moreover, IL2RG expression was negatively correlated with CD8+ T cells, macrophage M1, and activated memory CD4+ T cells.

**Figure 8:**
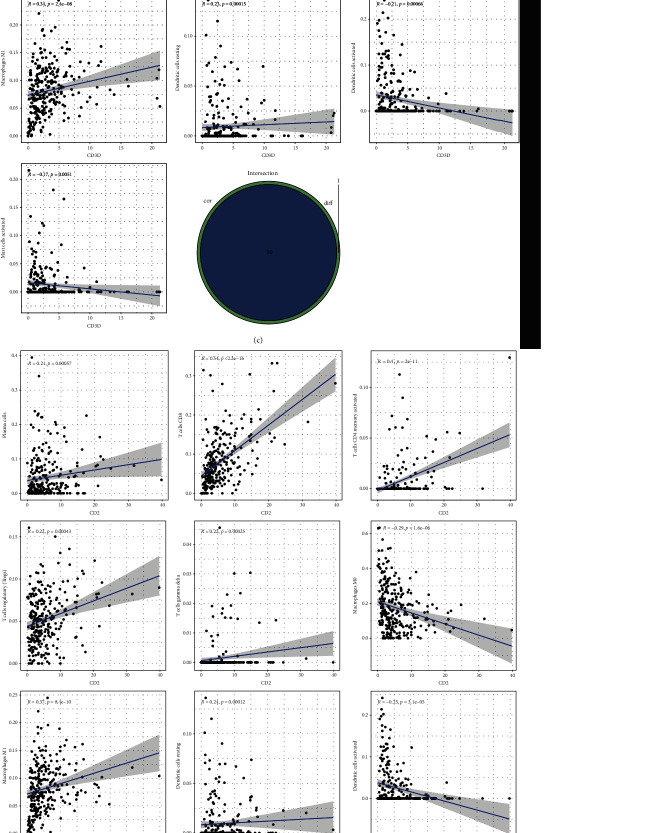
(a) Scatter plots illustrating the correlation between CD3E expression and 11 TIC-type proportions (*p* < 0.05). Each plot's blue line represents a fitted linear model that shows the immune cell's proportional tropism and CD3E expression. For correlation testing, the Pearson coefficient was used. Using difference and correlation analyses on violin and scatter plots, 11 TIC types are shown to be correlated with CD3E expression. (b) Scatter plots illustrating the correlation between CD3G expression and 12 TIC-type proportions (*p* < 0.05). Each plot's blue line represents a fitted linear model that shows the immune cell's proportional tropism and CD3G expression. For correlation testing, the Pearson coefficient was used. Using difference and correlation analyses on violin and scatter plots, 10 TIC types are shown to be correlated with CD3G expression. (c) Scatter plots illustrating the correlation between CD3D expression and 11 TIC-type proportions (*p* < 0.05). Each plot's blue line represents a fitted linear model that shows the immune cell's proportional tropism and CD3D expression. For correlation testing, the Pearson coefficient was used. By means of difference and correlation analyses on violin and scatter plots, 10 TIC types are shown to be correlated with CD3D expression. (d) Scatter plots illustrating the correlation between CD2 expression and 12 TIC-type proportions (*p* < 0.05). Each plot's blue line represents a fitted linear model that shows the immune cell's proportional tropism and CD2 expression. For correlation testing, the Pearson coefficient was used. Using difference and correlation analyses on violin and scatter plots, 11 TIC types are shown to be correlated with CD2 expression. (e) Scatter plots illustrating the correlation between CCL19 expression and 11-TIC type proportions (*p* < 0.05). Each plot's blue line represents a fitted linear model that shows the immune cell's proportional tropism and CCL19 expression. For correlation testing, the Pearson coefficient was used. Using difference and correlation analyses on violin and scatter plots, 10 TIC types are shown to be correlated with CCL19 expression. (f) Scatter plots illustrating the correlation between IL2RG expression and the proportion of 12 TIC types (*p* < 0.05). Each plot's blue line represents a fitted linear model that shows the immune cell's proportional tropism and IL2RG expression. For correlation testing, the Pearson coefficient was used. By means of difference and correlation analyses on violin and scatter plots, 10 TIC types are shown to be correlated with IL2RG expression.

The results indicated that the six crucial prognostic gene expressions had a substantial effect on immune activity in the TME (Figure 8).

## 4. Discussion

Using TCGA databases, we obtained data on RNA sequencing, and the corresponding clinical outcomes of OC to evaluate the TME profile and ICB therapy efficacy in patients with OC. The TME's immune and stromal cells can regulate tumor growth by secreting signal molecules and extracellular matrix components. Bone marrow mesenchymal vascular endothelial, and fibroblast cells are examples of stromal cells. Moreover, these stromal cells also produce many protumorigenic factors that recruit more protumorigenic cells and tumors to the growing microenvironment. This process can promote some mechanisms such as invasion, proliferation, metastasis, and tumor angiogenesis [13]. ICB therapy is a monoclonal antibody-based therapy that acts as a tumor suppressor via the mechanism of modulating tumor cell–immune cell interactions and boosts T cell-mediated antitumor immunity [14]. Immune checkpoint inhibitors (ICIs) are novel drugs based on immunotherapy. Moreover, ICIs promote the body's natural tumor-killing response. Immune checkpoint inhibitors such as cytotoxic T lymphocyte-associated molecule-4 monoclonal antibodies and PD-1/PD-L1 inhibitors are currently approved for treating certain cancers. Patients with different types of malignant tumors can benefit from immune checkpoint inhibitor treatment [15–17]. One study showed that the therapeutic effect of ICIs was correlated with the composition of the TME [18]. The current study showed that if the stromal cell composition is lower and the tumor purity is higher in the TME in patients with OC, ICB treatments can have better therapeutic effects. Therefore, investigating immune cell infiltration and gene expression in the OC microenvironment can provide novel ideas for identifying new targets for immunotherapy and for enhancing the efficacy of immunotherapy.

In the OC microenvironment, this study evaluated DEGs in immune and stromal cell components. We performed GO and KEGG enrichment analyses. The findings showed that these DEGs were primarily enriched in immune activity-related functions and pathways. Tumor cells can change the TME by producing some molecules that inhibit immune cells, thereby leading to immune system tolerance to the development of tumors and promoting tumor growth and metastasis. In this study, DEGs were linked to T-cell activation, lymphocyte and monocyte differentiation, and adhesion regulation between cells. Therefore, these DEGs changed the TME by affecting immune system activation and OC antitumor immune response. This further confirms TME's impact on OC prognosis.

CD3E, CD3G, CD2, CD3D, CCL19, and IL2RG were screened out from DEGs. Based on the survival analysis, patients with OC who had higher levels of these six genes had a better prognostic status. T cell receptor mediates antigen-induced signaling with the help of CD3E, CD3D, and CD3G, which participate in the conduction of antigen presentation signal [19]. In the development and organization of the immunological synapse, CD2 is crucial. CD2 also activates memory T cells and regulates NK cell activation [20]. CC chemokine ligand 19 (CCL19) is crucial in regulating immune responses. The CCL19 gene encodes the chemokine (C–C motif) ligand 19. Moreover, CCR7 and its ligands CCL19 and CCL21 participate in the recirculation of lymphocytes through secondary lymphoid organs [21]. Chemokines have a chemotactic affinity for immune cells and strong vascular inhibition and have attracted significant attention in the tumor immunotherapy [22]. CCL19 can enhance tumor T cell and dendritic cell-infiltration levels and PD-1/PD-L1 inhibitors' therapeutic effects [23]. Moreover, if T cells are activated, CCL19 can help T cells and dendritic cells home to lymphoid tissue's T cell region. Previous studies have shown that CCL19 may be used as a potential immune stimulant in immunotherapy for certain cancer types, including breast and lung cancers [24, 25]. CCL19 can substantially inhibit ovarian tumor growth and prolong survival after immunotherapy [26]. The Interleukin-2 (IL-2) receptor *γ* chain (IL2RG) gene encodes a protein that functions as a common receptor subunit for several important immune factors. This glycoprotein is found on the surface of most lymphocytes and aids the immune system. After its deletion, numerous immune functions are impaired. Importantly, the NK cell activity is completely lost [27, 28]. IL-2, IL-4, IL-7, IL-9, IL-15, and IL-21 comprise the cytokine family, which is the third receptor component of the IL-2 receptor based on their common initially identified cytokine receptor *γ* chain (*γ* C). They belong to the IL2RG family of cytokines, which is widely used in immunotherapy [29, 30]. In conclusion, the proteins encoded by CD3E, CD3G, CD2, CD3D, CCL19, and IL2RG are correlated with the immune cells. This then reveals the internal mechanism of their antitumor immune response, and their expression linked to prognosis in patients with OC. Therefore, GSEA enrichment analysis was conducted based on the expression of these six genes. The findings revealed that the genes with a high expression were primarily enriched in immunoactivity-related pathways. Therefore, they could influence immune cell infiltration in the TME, affecting the prognosis of patients with OC.

In OC, *γδ* T cell proportions correlated positively with CD3E, CD3G, CD2, CD3D, CCL19, and IL2RG expression. *γδ* T cells are T cells that have an innate immune function. They can kill cancer cells and tumor stem cells and can recognize cancer antigens. *γδ* T cells interact with the different immune cells, engage in antitumor immune responses, and are crucial in preventing tumor progression and inducing tumor cell apoptosis [31]. Previous research has illustrated that activating and improving *γδ* T cells cytotoxicity can improve the antitumor effects and the efficacy of tumor immunotherapy [32]. Moreover, *γδ* T cells can recognize isoprene pyrophosphate, which specifically identifies and attacks cancer cells [33]. *γδ* T cell immunotherapy, a novel tumor immunotherapy, has good clinical effects against different types of tumors, particularly malignant ones [34, 35]. Some studies have shown that the zoledronic acid-amplified *γδ* T cell transfer therapy is a safe and effective cure for patients with non-small cell lung cancer [36]. Its mechanism may be correlated with changes in the TME, which can attenuate *γδ*T cell response via different methods. Tumor-infiltrating *γδ* T cells in high-grade gliomas have a high-apoptosis rate, and the number of *γδ* T cells decreases significantly at the end stage of the disease [37, 38]. However, the TME's primary cytotoxic *γδ* T cell promoters, IL-2 and IL-15, are crucial for regulating cytotoxic *γδ* T cells in cancer immunotherapy [39]. Previous research has demonstrated that *γδ* T cells in OC have impaired antitumor cytotoxicity and enhanced immunosuppressive function, which can limit antitumor immunity, prevent immune surveillance, and promote OC progression [40]. Enhancing *γδ* T cell antitumor cytotoxicity within the TME and strengthening the antitumor immune response can inhibit OC progression and improve prognosis in patients with OC. Immunotherapy based on *γδ* T cells has several advantages. However, there is no current in-depth research about the clinical application of such treatment. *γδ* T cell reduction in the TME can be a prognostic predictor in patients with OC. Moreover, optimizing *γδ* T cell immunotherapy and improving its clinical efficacy can help in enhancing patient prognosis.

In conclusion, CD3E, CD3G, CD2, CD3D, CCL19, and IL2RG were screened using bioinformatics methods. These genes were involved in the immune-related molecular mechanisms and could regulate TICs. Their expression was positively correlated with *γδ* T cells. Therefore, they can be potential prognostic markers and immunotherapy targets in the OC microenvironment. This study can provide novel ideas regarding the application of OC immunotherapy. However, the results are only based on bioinformatics analysis. Nevertheless, the role of the abovementioned genes should be validated via cell and animal experiments, and the specific molecular mechanism must be further explored.

## Data Availability

The data used to support the findings of this study are available from the corresponding author upon reasonable request.

## Abbreviations

BP: Biological process
CC: Cell component
ACT: Cell therapy
CTLs: Cytotoxic T lymphocytes
DEGs: Differentially expressed genes
FDR: False discovery rate
GO: Gene ontology
GSEA: Gene set enrichment analysis
ICB: Immune checkpoint blockade
IL-2: Interleukin-2
IL2RG: Interleukin-2 receptor *γ* chain
KEGG: Kyoto Encyclopedia of Genes and Genomes
MF: Molecular function
OC: Ovarian cancer
PPI: Protein–protein interaction
TCGA: The Cancer Genome Atlas
TIDE: The tumor immune dysfunction and exclusion
TICs: Tumor-infiltrating immune cells
TME: Tumor microenvironment.
